# Correction: Detection of novel coronaviruses in bats in Myanmar

**DOI:** 10.1371/journal.pone.0295490

**Published:** 2023-12-04

**Authors:** Marc T. Valitutto, Ohnmar Aung, Kyaw Yan Naing Tun, Megan E. Vodzak, Dawn Zimmerman, Jennifer H. Yu, Ye Tun Win, Min Thein Maw, Wai Zin Thein, Htay Htay Win, Jasjeet Dhanota, Victoria Ontiveros, Brett Smith, Alexandre Tremeau-Bravard, Tracey Goldstein, Christine K. Johnson, Suzan Murray, Jonna Mazet

The fifteenth author’s name is spelled incorrectly. The correct name is: Alexandre Tremeau-Bravard.

There is an error in the corresponding author’s email address. The correct email address is: valitutto@ecohealthalliance.org.
