# Supplementary material for: Detection of novel coronaviruses in bats in Myanmar
Source: PLoS One. 2020 Apr 9;15(4):e0230802. doi: 10.1371/journal.pone.0230802 (PMC7144984; doi:10.1371/journal.pone.0230802)
Supplement: Attachment — Submitted filename: Response to Reviewers_Valitutto et al..docx [file pone.0230802.s003.docx]

**Responses to Reviewers** (Comments to the Author)

**Reviewer #1:**

I have uploaded my comments on the manuscript for the author to review and take into consideration when re-writing the manuscript. Importantly the phylogenetic tree to show the relationship between the corona viruses.

- Line 65 needs literature quotation
- Lines 68-70 need literature quotation
- Lines 90-92 need literature quotation

***Thank you for finding these errors. The manuscript has been revised to include literature citations for these lines listed.***

- Line 110 Animal capture and sampling: General comment is to clarify to the reader if there was any form of anesthesia application on the big chiropterans that couldn’t be handled manually during sampling.

***Thank you for identifying this omission. All animals were handled manually; no anesthetic or immobilization agents were used to assist with capture or restraint, and the manuscript has been corrected to reflect this.***

- Also Line 131-132: after how long were the bats released after capture and sampling. Time estimate here would be good for the readers.

***Thank you for the suggestion. We have noted in the manuscript the following text: “Bats were released within 1 km of the capture site as soon as possible upon completion of each sampling event, with net capture and pillowcase restraint between 5 to 30 minutes and handling times less than 5 minutes for each individual.”***

- Line 162 Results general comment is mainly on seasonality. Can the season/months be indicated in the table of results as it is stated at length in the results section.

***Thank you for this very reasonable suggestion. However, the authors note that seasonality was intentionally discussed at greater length in the results of the main body of the paper, as the authors did not find a way to naturally insert the data into Table 1, given that the species captured and sites spanned both dry- and wet-seasons. Results not easily depicted in the table were discussed at greater length in the main body.***

- Still in the results section; - a phylogenetic tree is needed to indicate to the reader the relationship between the beta and alpha corona viruses with the known and unknown corona viruses.

***Thank you for the suggestion. However, the authors note that only short fragments of the RdRp gene (328bp and 434 bp, depending on the assay used) were amplified. Given the short sequence fragment of a conserved gene, phylogenetic analyses would likely be uninformative and limited in value.*** ***The authors agree that this is a shortcoming of the paper. However, the purpose of the study was not to fully characterize specific viruses, but to broadly screen for viruses in wildlife (especially bats) living in close proximity to human communities, and thus to better understand potential sources of zoonotic viral transmission in the context of these human-wildlife interfaces. The discussion has been expanded to further discuss this.***

- Line 178 Since you used cyt B for species identification: why are the four host species of the pooled guano sampled aren’t identified? The reader might wonder.

***Thank you very much for identifying this error! It has come to the attention of the authors that the initial table and figure were created with an older version of the data, without the final host species barcoding incorporated. This has been corrected in the body of the manuscript, in Table 1, and in Figure 1. As such, there are no positive samples where the host species was not identified by barcoding. The four pooled guano samples referred to in Line 178 were attributed to* Chaerephon plicatus*.***

**Reviewer #2:**

- In the Methods section, it is not clear how much of the initial sample volume used for RNA extraction from each of the oral, rectal and guano samples. Although one would expect, this is specially important since the oral swabs yielded the lowest.

***The authors agree with this assessment. The manuscript has been revised to reflect our methodology: samples were collected in 500 µl Trizol, and 250 µl was used from each sample for extraction. One aliquot was used for extraction per the RNA extraction kit, and an additional aliquot was stored to ensure that sufficient volume was available for a second extraction, or other downstream analyses if needed. 8 µl RNA (i.e. the maximum volume for the Superscript III kit) was used for cDNA transcription.***

- Additionally, if deposited in GenBank or elsewhere the sequence data accession numbers need to be provided and mentioned here.

***The authors are in agreement with the reviewer. The sequence* *data have been uploaded to NCBI GenBank since submission of the first draft of the manuscript, and the accession numbers have been added in a supplemental table. At the time of resubmission, the sequences have not yet been published.***

- In the Results and discussion, Authors need to indicate if at all any virus culture been attempted and if any of these viruses has been isolated and characterized.

***Thank you for identifying this omission. The authors have corrected the manuscript to reflect that viral culture and isolation were not pursued in this study.***

- The absence of at least RdRp partial sequence phylogenetic analysis is a shortcoming for this report that needs to be discussed or addressed. On a similar note, a phylogenetic analysis derived from spike(S) and receptor-binding domain (RBD) would be informative to include here. Moreover, this report could be further improved if they consider providing information on the protein sequence alignment of coronavirus RdRps or S proteins with the conserved motifs and other unique signatures indicated as necessary.

***Thank you for the suggestions. The authors note that only short fragments of the RdRp gene (328bp and 434 bp, depending on the assay used) were amplified. Given the short sequence fragment of a conserved gene, protein sequence analysis would not be informative. This is the same reason why a phylogenetic analysis was not pursued. The authors agree that this is a shortcoming of the paper. However, the purpose of the study was not to fully characterize specific viruses, but to broadly screen for viruses in wildlife (especially bats) living in close proximity to human communities, and thus to better understand potential sources of zoonotic viral transmission in the context of these human-wildlife interfaces. The discussion has been expanded to further discuss this.***

- Although their limited sequencing results may not allow, if the novel viruses found in this study would constitute recent recombination events from existing coronaviruses need to be at least discussed since such recombination is rather common and are thought to contribute to the emergence of novel coronaviruses.

***Thank you very much for the suggestion. However, the authors note that it is not possible to assess recombination from a short fragment of the RdRp gene as was performed in this study; thus, those analyses were not performed and therefore not included in the discussion. We agree that should complete genomes have been amplified, or the complete spike gene sequence amplified, those analyses to identify recombination and associated discussion may have been appropriate. The discussion has been expanded to further discuss this.***
