# Supplementary material for: Detection of novel coronaviruses in bats in Myanmar
Source: PLoS One. 2020 Apr 9;15(4):e0230802. doi: 10.1371/journal.pone.0230802 (PMC7144984; doi:10.1371/journal.pone.0230802)
Supplement: Attachment — Submitted filename: Reviewer Comments.docx [file pone.0230802.s002.docx]

**Comments**

This is a very good paper highlighting the corona viruses detection in Myanmar. Important to understand the role of bats in corona viruses ecology for the country and the region.

These are my comments: -

Line 65 needs literature quotation

Lines 68-70 need literature quotation

Lines 90-92 need literature quotation

Line 110 Animal capture and sampling: General comment is to clarify to the reader if there was any form of anesthesia application on the big chiropterans that couldn’t be handled manually during sampling.

Also Line 131-132: after how long were the bats released after capture and sampling. Time estimate here would be good for the readers.

Line 162 Results general comment is mainly on seasonality. Can the season/months be indicated in the table of results as it is stated at length in the results section.

Still in the results section; - a phylogenetic tree is needed to indicate to the reader the relationship between the beta and alpha corona viruses with the known and unknown corona viruses.

Line 178 Since you used cyt B for species identification: why are the four host species of the pooled guano sampled aren’t identified? The reader might wonder.
